# Bowhead: Bayesian modelling of cell velocity during concerted cell migration

**DOI:** 10.1371/journal.pcbi.1005900

**Published:** 2018-01-08

**Authors:** Mathias Engel, James Longden, Jesper Ferkinghoff-Borg, Xavier Robin, Gaye Saginc, Rune Linding

**Affiliations:** 1 Biotech Research and Innovation Centre, University of Copenhagen, Copenhagen, Denmark; 2 Niels Bohr Institute, University of Copenhagen, Copenhagen, Denmark; Universite de Montreal, CANADA

## Abstract

Cell migration is a central biological process that requires fine coordination of molecular events in time and space. A deregulation of the migratory phenotype is also associated with pathological conditions including cancer where cell motility has a causal role in tumor spreading and metastasis formation. Thus cell migration is of critical and strategic importance across the complex disease spectrum as well as for the basic understanding of cell phenotype. Experimental studies of the migration of cells in monolayers are often conducted with ‘wound healing’ assays. Analysis of these assays has traditionally relied on how the wound area changes over time. However this method does not take into account the shape of the wound. Given the many options for creating a wound healing assay and the fact that wound shape invariably changes as cells migrate this is a significant flaw. Here we present a novel software package for analyzing concerted cell velocity in wound healing assays. Our method encompasses a wound detection algorithm based on cell confluency thresholding and employs a Bayesian approach in order to estimate concerted cell velocity with an associated likelihood. We have applied this method to study the effect of siRNA knockdown on the migration of a breast cancer cell line and demonstrate that cell velocity can track wound healing independently of wound shape and provides a more robust quantification with significantly higher signal to noise ratios than conventional analyses of wound area. The software presented here will enable other researchers in any field of cell biology to quantitatively analyze and track live cell migratory processes and is therefore expected to have a significant impact on the study of cell migration, including cancer relevant processes. Installation instructions, documentation and source code can be found at http://bowhead.lindinglab.science licensed under GPLv3.

This is a *PLOS Computational Biology* Software paper.

## Introduction

The coordinated movement of cells is required for almost any morphogenetic processes [1] and plays a key role in biological organization [2]. Deregulation of the migratory response is known to be associated with various pathological conditions including vascular disease, chronic inflammatory diseases, mental retardation and cancer, where cell motility plays a causal role in tumor invasion and metastasis [3]. Greater knowledge on the role of signaling networks in cell migration is therefore likely to lead to the identification of novel therapeutic targets, in addition to a general improvement in our basic scientific understanding of this emergent property.

The migration of cells in monolayers is commonly studied using a ‘scratch assay’, where a wound is created by scratching a confluent layer of cells [4], or the derived zone exclusion assay where a wound is created using a physical, removable barrier which prevents cells from becoming confluent [5]. In both cases, analysis of the migration of cells into the wound, referred to as ‘wound healing’, commonly focuses on the change in open wound area [6] or the slope of the area change [7]. However, these methods are fundamentally flawed given that they ignore the effect of wound geometry i.e. the shape of the wound space.

**Fig 1:**
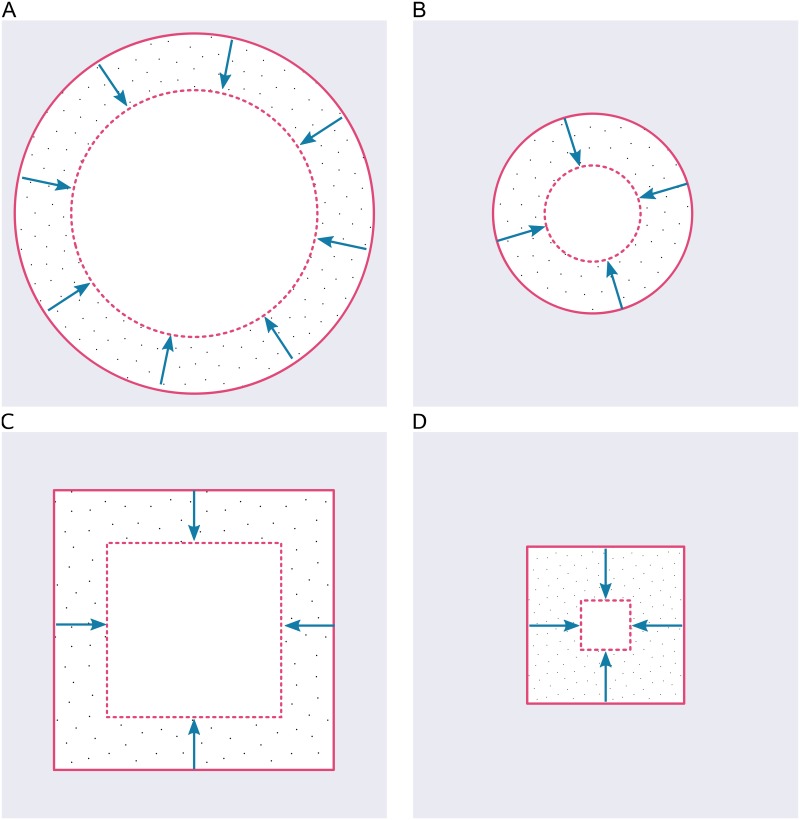
Effect of wound geometry on decreasing wound area. Cells moving at a constant velocity can cause significantly different changes in wound area depending on the initial perimeter as illustrated in the following examples: The wound shown in (A) decreases in area by 48%. The wound in (B) has the same shape as in (A) and a smaller perimeter, and decreases in area by 75%. Similarly, the wound in (C) has the same perimeter length as in (A) but a different shape and decreases in area by 58%. Finally the wound in (D) is the same shape as in (C) and a smaller perimeter, and decreases in area by 86%. The velocity of the moving cells is identical in all cases.

Here we argue that a more accurate representation of wound closure is the concerted cell velocity derived from wound area and perimeter, as shown in Fig 1. We have developed a computational method for identifying wounds in images with labeled cells, calculating the area and perimeter of the identified wounds and from these measurements determining the velocity using a unique Bayesian statistical approach. Bayesian statistics is increasingly used in many fields as it allows the generation of inferences from observed data where those inferences are dependent on uncertain parameters or missing data [8]. In practical terms our algorithm applies a Gaussian Process Regression [9] to the determined wound area and perimeter measurements in order to derive probability distributions of velocities over all possible time points, both measured and unmeasured.

As such we believe that our wound detection algorithm can address a key deficiency in currently available tools. Given that there is no standardized experimental method for creating wounds in a scratch assay quantifying cell velocity would also allow for better reproducibility of experimental data between laboratories, given that cell velocity is independent of wound geometry. We demonstrate the use of our algorithm in a study of migration in a cancer cell model, and using the TScratch sample data [6]. For both data sets we show that measuring cell velocity can increase assay quality compared to measuring changes in wound area.

## Design and implementation

The Bowhead cell velocity method was implemented as an open source Python package with methods to detect, fit and predict concerted cell velocity. The program can analyze any type of image that fits, or can be processed to fit, the general assumption that cell regions have higher intensity counts than wound regions. The program structure facilitates easy incorporation into imaging pipelines for use in screening assays. For each time point analyzed by the package wound perimeter and area were quantified. Measurement variance could then be estimated by repeating the detection at slightly different thresholding values in order to mimic the inherent uncertainty of the wound boundary.

The detection algorithm, shown in S1 Fig, convoluted each image with a two-dimensional Gaussian kernel with standard deviation *σ*. This convolution was used to reduce noise smaller than the biologically relevant length scale. Confluency intensity was defined per image as

$$\begin{array}{c} I_{c}=\frac{\sum_{i=1}^{N}p_{i}^{2}}{\sum_{i=1}^{N}p_{i}}, \end{array}$$ (1)

where *p*_*i*_ was the intensity value of a pixel at position *i* and *N* was the total number of pixels in the image.

Given a user defined relative intensity scaling factor *β* ∈ ]0, 1[ the convoluted image was binarized at an absolute threshold of *βI*_*c*_. The largest connected region of pixels, in a Von Neumann neighbourhood, below this threshold was then classified as the wound. The unclassified area was then filled such that its topology was simply connected. Wound area could then be defined by

$$\begin{array}{c} w_{\alpha }=\sum_{i=1}^{N}d\left(p_{i}\right),\;d\left(p_{i}\right)=\{\begin{array}{cc} 0 & p_{i}\notin R\\ 1 & p_{i}\in R \end{array}, \end{array}$$ (2)

where *N* was the total number of pixels in the image and *R* is the wound region.

To determine the wound perimeter the wound region was traced with the Marching Squares algorithm [10] (S1 Fig), with zero-padding, to ensure the detection of a closed contour *S*. The euclidian length of *S* excluding image border *B* thus defined the wound perimeter

$$\begin{array}{c} w_{\phi }=\int_{C}S\;ds-\sum_{n=1}^{N_{S}}b\left(p_{i}\right),\;b\left(p_{i}\right)=\{\begin{array}{cc} 0 & p_{i}\notin B\\ 1 & p_{i}\in B \end{array}, \end{array}$$ (3)

where *N*_*S*_ was the number of pixels in *S*. Bowhead also contains an 8-direction chain code algorithm [11] for determining perimeters that is limited to making turns at whole pixels (the Marching Squares algorithm can make turns at sub-pixel resolution due to interpolation). This gave a perimeter that was slightly less accurate (S2 Fig), but in a shorter processing time.

In order to limit the detection of erroneous wounds a reference coordinate **p** was defined from the wound’s center of mass *x*(*t*) at the two first time points *t*_1_ and *t*_2_

$$\begin{array}{c} \text{p}=\frac{\text{x}\left(t_{1}\right)+\text{x}\left(t_{2}\right)}{2}. \end{array}$$ (4)

For subsequent time points only regions inside a radius *r* from **p** would be considered as wound candidates. This ensured that dark spots far from the wound, e.g. at the image corners, was not detected. The contour tracing was not affected by this parameter.

After wound detection, Gaussian Process Regression (GPR) [9] was used to model the wound area and perimeter as functions of time separately. The Gaussian process is fully represented by it’s mean and co-variance functions.

$$\begin{array}{cc} m(\text{t}) & =E[f(\text{t})], \end{array}$$ (5)

$$k(\text{t},{\text{t}}^{\prime })=E[f\left(\text{t}\right)-m\left(\text{t}\right)f\left({\text{t}}^{\prime }\right)-m\left({\text{t}}^{\prime }\right)],$$ (6)

where **t** and **t**′ are the different input time points, *f* the prediction funtion, *k* the covariance kernel and *m* the mean function. In short

$$f(\text{t})∼GP(m\left(\text{t}\right),k\left(\text{t},{\text{t}}^{\prime }\right))$$ (7)

**Fig 2:**
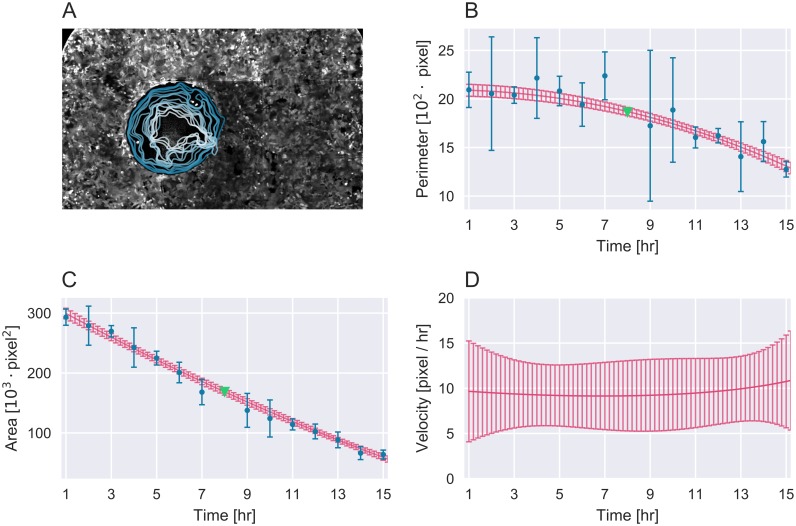
Analysis of a single wound healing experiment of MDA-MB-231 cells under POU5F1 siRNA knockdown. (A) Detected wound at 1 hour (blue line) progressing to 15 hours (white line). The algorithm utilizes a combination of measured data (blue) and predicted data (red) in order determine the change in wound perimeter (B), wound area (C) and the derived cell velocity (D). Expected data which is found to be missing can be imputed by the model (green). All error bars signify one standard deviation of data and predictions colored accordingly.

The mean function was zero since the GPR training was performed on the normalized time series. The sum of a constant and a homogeneous quadratic kernel [12] was used as variance priors for the Gaussian processes (GP). Two GPs were initialized and trained by optimizing the log likelihood expression in eq. 5.9 in [13]. This gave two GPs, trained with the measured area *w*_*α*_(**t**) and perimeter *w*_*ϕ*_(**t**) respectively

$$w_{\alpha }\left(\text{t}\right)∼GP(0,C_{\alpha }+{\left(\sigma_{\alpha }^{2}+\text{t}\cdot {\text{t}}^{\prime }\right)}^{2})$$ (8)

$$w_{\phi }\left(\text{t}\right)∼GP(0,C_{\phi }+{\left(\sigma_{\phi }^{2}+\text{t}\cdot {\text{t}}^{\prime }\right)}^{2})$$ (9)

where *C*_*α*_ and *C*_*ϕ*_ are constants. The fitted GPs allow for posterior predictions with Bayesian estimated uncertainty of area and perimeter (red lines Fig 2B and 2C), weighting data detection uncertainty by the prior kernel. By using a Bayesian model it was possible to impute missing values with uncertainty. The kernel learning approach allowed any arbitrary time curve shape, restricted only by the prior and the observed data. From the GP predicted wound area, perimeter and uncertainties cell velocity could then be defined as

$$\begin{array}{c} v\left(t\right)=-\frac{\frac{d}{dt}{\hat{w}}_{\alpha }\left(t\right)}{{\hat{w}}_{\phi }\left(t\right)}, \end{array}$$ (10)

where ${\hat{w}}_{\alpha }\left(t\right)$ and ${\hat{w}}_{\phi }\left(t\right)$ were the predicted time dependent area and perimeter respectively. $\frac{d}{dt}{\hat{w}}_{\alpha }\left(t\right)$ was found numerically using the second order central difference method. The standard deviation of the velocity was derived through standard error propagation.

## Results

Using our method we investigated the concerted velocity of MDA-MB-231 cells, an epithelial-like breast cancer cell line with a very strong migrating phenotype [14], in a zone exclusion assay. We performed a literature search to identify proteins that have been reported to affect the migratory response of MDA-MB-231 cells and selected three of these to validate our method. We chose myosin heavy chain 9 (MYH9) and POU class 5 homeobox 1 (POU5F1), which were expected to increase and decrease migration respectively upon siRNA knockdown ([15] [16] [17] [18]). Finally we chose polo-like kinase 1 (PLK1) as a ‘no migration’ control. PLK1 dysfunctionality is reported to cause apoptosis of cells due to its crucial role in establishment of the bipolar spindle during mitosis [19] [20]. We therefore hypothesised that siRNA knockdown of PLK1 would lead to cells unable to migrate or proliferate. S3 Fig shows images of wounds with PLK1 knockdown compared to the other conditions confirming the expected non-migratory phenotype.

Cells were transfected with pooled Silencer Select siRNAs (three siRNAs per pool, 10nM final concentration) using lipofectamine RNAiMAX for 48 hours prior to experimentation. In addition to the three siRNAs previously described cells were also transfected with a non-targeting siRNA, which should represent the migratory potential of unperturbed MDA-MB-231 cells. Following gene knockdown the zone exclusion was removed and cells were imaged for 16 hours in 1 hour intervals on a high content screening system (PerkinElmer Opera) at 10x magnification (1.3μm per pixel resolution) to detect red fluorescent protein tagged histone (nuclear staining) and diffuse green fluorescent protein (cytoplasmic staining).

Each of the four knockdowns was repeated 28 times generating 112 time series in total. The exclusion wounds were detected using the following settings, *σ* = 18, *β* = 0.3 ± 0.08, *r* = 100 on the cytoplasmic image channel.

Fig 2A and S1 Video show an example wound detection result from this experiment and illustrate the reliable detection of wounds in noisy lighting conditions, and the accurate tracing of perimeters even with drifting cells inside the wound zone (also, see S2 Video for an example of a scratch assay detection). For each time series the wound was considered closed when its area had shrunk to below 5‰ of the image area. Any remaining time points were thus excluded from further analysis. Area (Fig 2B) and perimeter (Fig 2C) detection data was used to train velocity models for all replicas. As expected, due to the geometric effect illustrated in Fig 1, we found that the area and perimeter do not change linearly with time but curve slightly in opposite concavity. The concerted cell velocity was derived and as illustrated in Fig 2D it was found to be constant over time. The predicted velocity with uncertainty reflects the variance of the measured wound data and takes missing data into account. The Bayesian modelling allowed for imputation of missing data and uncertainty (Fig 2D).

**Fig 3:**
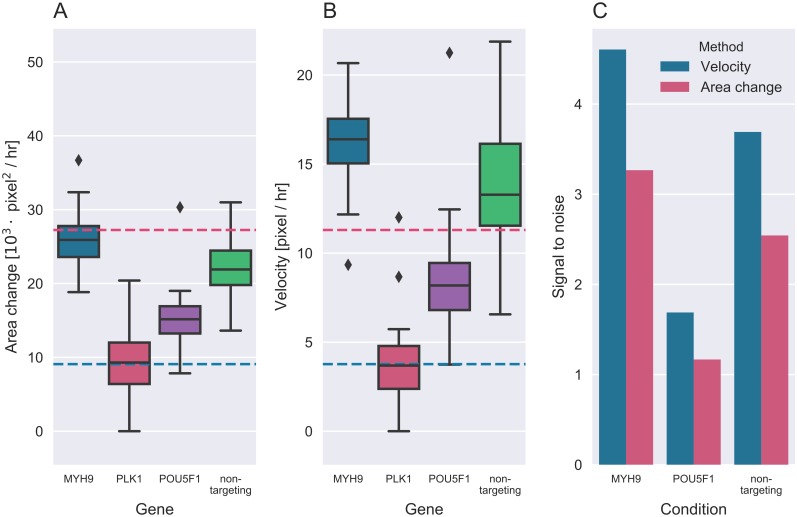
Comparison of mean velocity and area change for wound healing experiments with 3 different siRNA knockdowns. (A) Mean area change. (B) Mean velocity. Blue lines illustrate PLK1 mean response and red lines illustrate 3 times PLK1 response. (C) Signal to noise comparison of the area change and mean velocity.

To compare the computed cell velocities with the more conventional readout of change in area over time the mean change of area was found by taking the slope of a linear least square regression from the area data for each time series. For velocity the weighted average of the velocity was calculated for each time series weighted by the velocity precision $\sigma_{v}^{-2}$. For both velocity and area change we computed the group mean and standard deviation for each experimental condition (Fig 3). Cell velocity was found to increase, compared to non-targeting siRNA, upon MYH9 knockdown and decrease after knockdown of POU5F1 and PLK1 as expected. The order from slow to fast of the various perturbations was found to match qualitative assessment of velocity from images as shown in S2 Fig. Cell velocity was found to give a larger assay window compared to change in area as shown in Fig 3. Cells treated with siRNAs against MYH9 and non-targeting siRNAs were found to have mean velocities greater than three times the mean of cells treated with PLK1 siRNA. None of the perturbations showed this effect when quantifying change in wound area. To further illustrate and quantify this difference in condition spread we calculated the signal to noise ratio using PLK1 values as the base line

$$\begin{array}{c} \text{SNR}=\frac{\mu_{\text{cond.}}-\mu_{\text{PLK1}}}{\sigma_{\text{PLK1}}}. \end{array}$$

**Fig 4:**
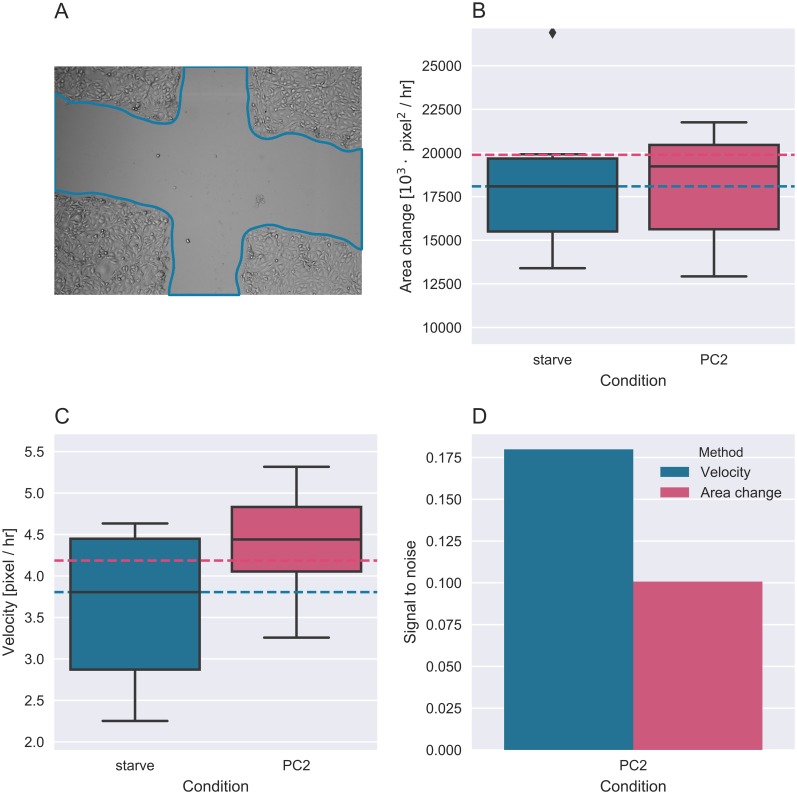
Comparison of mean velocity and area change for TScratch sample data. (A) Wound detection in non-fluorescent images. (B) Mean area change. (C) Mean velocity. Blue lines illustrate starve response median value and red lines illustrate 110% of this value. (D) Signal to noise comparison of the area change and mean velocity.

Fig 3C shows SNR for cells treated with MYH9, POU5F1 and non-targeting siRNAs. In all cases concerted velocity was found to outperform the current method of area change, giving increased dynamic range. Similarly, sample data supplied with the TScratch algorithm was also analyzed using Bowhead. As these images were not fluorescent the images were first preprocessed using a Scharr gradient filter to facilitate the accurate detection of wounds. Again velocity better separated the culture conditions assayed in the TScratch sample data, almost doubling the signal to noise ratio Fig 4.

These analyses demonstrate that determining cell velocity enables better separation of conditions in high content screening assays and would thus facilitate more accurate detection of potential therapeutic targets or treatment strategies. We note that wound geometry can change over time for no obvious biological reason (see imaging data associated with this paper on bowhead.lindinglab.science for examples), wounds that are circular to begin with become more square or triangular over time. In these cases the imaging method, mainly the number of time points used to calculate the wound closure, would have a significant impact on the result if area change was used, and may also affect the signal-to-noise ratio. In comparison, velocity is an absolute measurement that was not affected by the different imaging methods, and instrumentation, employed to generate the two datasets tested here.

## Availability and future directions

For platform independence the wound detection algorithm was constructed using Python. Documentation, source code, data used in the paper and examples are hosted online at http://bowhead.lindinglab.science. The package can be installed directly with the terminal command ‘pip3 install bowhead’ (via http://pypi.org). The work is licensed under GPLv3. The presented method quantifies dynamic information on cell velocity. Our focus has been on generating accurate wound detection for high throughput analysis of fluorescent images. The tool described also has a setting to work on bright field and phase-contrast images but could be developed further to provide more accurate wound detection when signal to noise ratios of pixel intensities are low. We will therefore pursue the use and development of this software in experiments where it is expected that cells alter speed during measurement in response to conditions such at cell damage stress or delayed drug response.

## Supporting information

S1 Fig Steps of the wound detection algorithm. The original image (A) is first flattened to gray scale, then convoluted with a Gaussian filter at chosen standard deviation *σ* (B), binarized (C) and finally the wound contour is traced with Marching Squares algorithm (D). The contour is traced at multiple thresholds to estimate uncertainty in the wound detection.
(TIF)

S2 Fig Comparison of wound perimeters generated by Marching Square and Chain Code algorithms. Both are available with Bowhead.
(TIF)

S3 Fig Wound closing ability effected by siRNA knockdown. Cells with PLK1 knockdown are not migrating. MYH9 and POU5F1 knockdown cells are migrating faster and slower respectively compared to non-targeting cells.
(TIF)

S1 Video Wound detection on an exclusion zone assay. Non-targeting cells migrating into the wound with detected border in blue, 1 to 11 hours.
(MP4)

S2 Video Wound detection on a scratch assay. Non-targeting cells migrating into the wound with detected border in blue, 1 to 17 hours.
(MP4)

## References

[1] Ridley A, Schwartz M, Burridge K, Firtel R, Ginsberg M, Borisy G, et al Cell Migration: Integrating Signals from Front to Back. Science. 2003;302(5651):1704–1709. doi: 10.1126/science.1092053 14657486 10.1126/science.1092053

[2] Lauffenburger DA, Horwitz AF. Cell Migration: A Physically Integrated Molecular Process. Cell. 1996;84(3):359–369. http://doi.org/10.1016/S0092-8674(00)81280-5. 8608589 10.1016/s0092-8674(00)81280-5

[3] Friedl P, Wolf K. Tumour-cell invasion and migration: diversity and escape mechanisms. Nat Rev Cancer. 2003;3(5):362–374. doi: 10.1038/nrc1075 12724734 10.1038/nrc1075

[4] Liang CC, Park AY, Guan JL. In vitro scratch assay: a convenient and inexpensive method for analysis of cell migration in vitro. Nat Protoc. 2007;2(2):329–33. doi: 10.1038/nprot.2007.30 17406593 10.1038/nprot.2007.30

[5] Hulkower KI, Herber RL. Cell migration and invasion assays as tools for drug discovery. Pharmaceutics. 2011;3(1):107–24. doi: 10.3390/pharmaceutics3010107 24310428 10.3390/pharmaceutics3010107 PMC3857040

[6] Gebäck T, Schulz MMP, Koumoutsakos P, Detmar M. TScratch: a novel and simple software tool for automated analysis of monolayer wound healing assays. Biotechniques. 2009;46(4):265–74. 19450233 10.2144/000113083

[7] Masuzzo P, Hulstaert N, Huyck L, Ampe C, Van Troys M, Martens L. CellMissy: a tool for management, storage and analysis of cell migration data produced in wound healing-like assays. Bioinformatics. 2013;29(20):2661 doi: 10.1093/bioinformatics/btt437 23918247 10.1093/bioinformatics/btt437 PMC3789541

[8] Eddy SR. What is Bayesian statistics? Nat Biotechnol. 2004;22(9):1177–8. 15340486 10.1038/nbt0904-1177

[9] Rasmussen CE, Williams KI. 2. In: Gaussian processes for machine learning. MIT Press; 2006.

[10] Lorensen WE, Cline HE. Marching cubes: A high resolution 3D surface construction algorithm. In: ACM siggraph computer graphics. vol. 21. ACM; 1987. p. 163–169.

[11] Myler HR, Weeks AR. The pocket handbook of image processing algorithms in C. Prentice Hall Press, 2009 32–34

[12] Rasmussen CE, Williams KI. 4. In: Gaussian processes for machine learning. MIT Press; 2006.

[13] Rasmussen CE, Williams KI. 5. In: Gaussian processes for machine learning. MIT Press; 2006.

[14] Price JE, Polyzos A, Zhang RD, Daniels LM. Tumorigenicity and metastasis of human breast carcinoma cell lines in nude mice. Cancer research. 1990;50(3):717–721. 2297709

[15] Even-Ram S, Doyle AD, Conti MA, Matsumoto K, Adelstein RS, Yamada KM. Myosin IIA regulates cell motility and actomyosin–microtubule crosstalk. Nature cell biology. 2007;9(3):299–309. doi: 10.1038/ncb1540 17310241 10.1038/ncb1540

[16] Schramek D, Sendoel A, Segal JP, Beronja S, Heller E, Oristian D, et al Direct in vivo RNAi screen unveils myosin IIa as a tumor suppressor of squamous cell carcinomas. Science. 2014;343(6168):309–13. doi: 10.1126/science.1248627 24436421 10.1126/science.1248627 PMC4159249

[17] Xin Yh, Yang Xj, Cui W, Cui Hj, Cui Yh, Zhang X, et al POU5F1 enhances the invasiveness of cancer stem-like cells in lung adenocarcinoma by upregulation of MMP-2 expression. PloS one. 2013;8(12):e83373 doi: 10.1371/journal.pone.0083373 24386189 10.1371/journal.pone.0083373 PMC3875455

[18] Kobayashi K, Takahashi H, Inoue A, Harada H, Toshimori S, Kobayashi Y, et al Oct-3/4 promotes migration and invasion of glioblastoma cells. Journal of cellular biochemistry. 2012;113(2):508–517. doi: 10.1002/jcb.23374 21938739 10.1002/jcb.23374

[19] Liu Z, Sun Q, Wang X. PLK1, A Potential Target for Cancer Therapy. Transl Oncol. 2017;10(1):22–32. doi: 10.1016/j.tranon.2016.10.003 27888710 10.1016/j.tranon.2016.10.003 PMC5124362

[20] Weiß L, Efferth T. Polo-like kinase 1 as target for cancer therapy. Experimental hematology & oncology. 2012;1(1):38 doi: 10.1186/2162-3619-1-38 23227884 10.1186/2162-3619-1-38 PMC3533518

