# Supplementary figures and images for: Bowhead: Bayesian modelling of cell velocity during concerted cell migration
Source: PLoS Comput Biol. 2018 Jan 8;14(1):e1005900. doi: 10.1371/journal.pcbi.1005900 (PMC5774831; doi:10.1371/journal.pcbi.1005900)

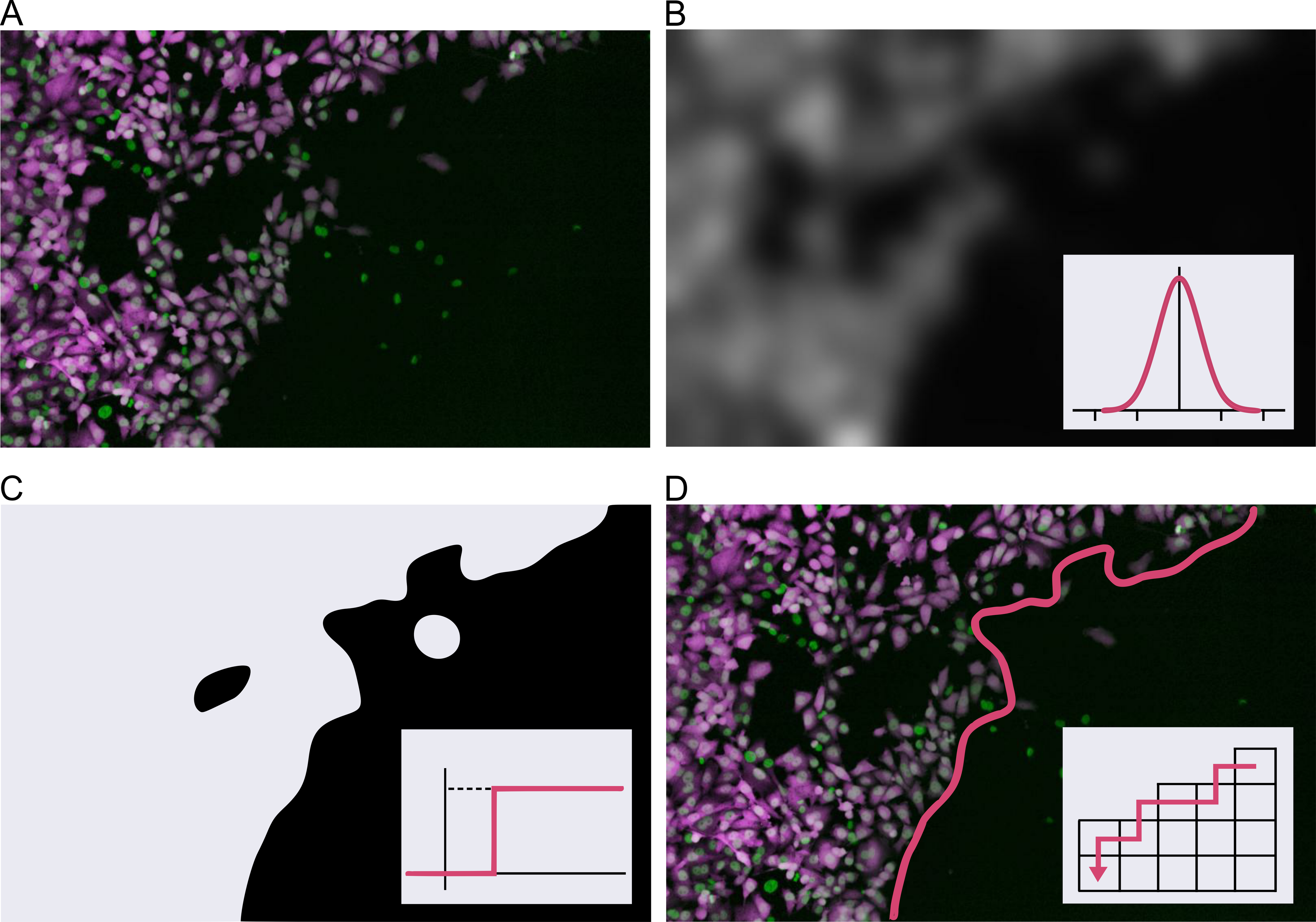

Supplement: S1 Fig — The original image (A) is first flattened to gray scale, then convoluted with a Gaussian filter at chosen standard deviation σ (B), binarized (C) and finally the wound contour is traced with Marching Squares algorithm (D). The contour is traced at multiple thresholds to estimate uncertainty in the wound detection. (TIF) [file pcbi.1005900.s001.tif]

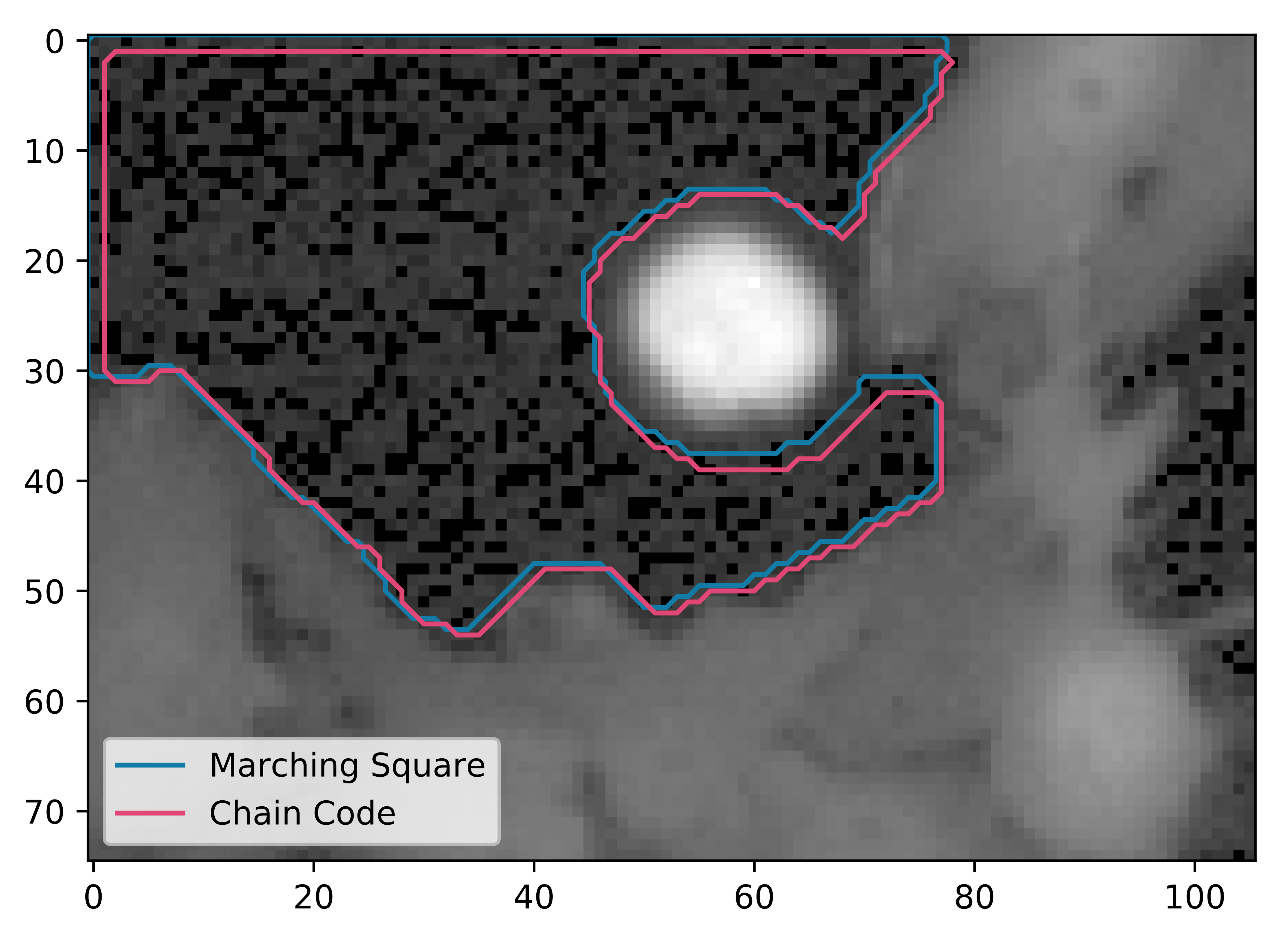

Supplement: S2 Fig — Both are available with Bowhead. (TIF) [file pcbi.1005900.s002.tif]

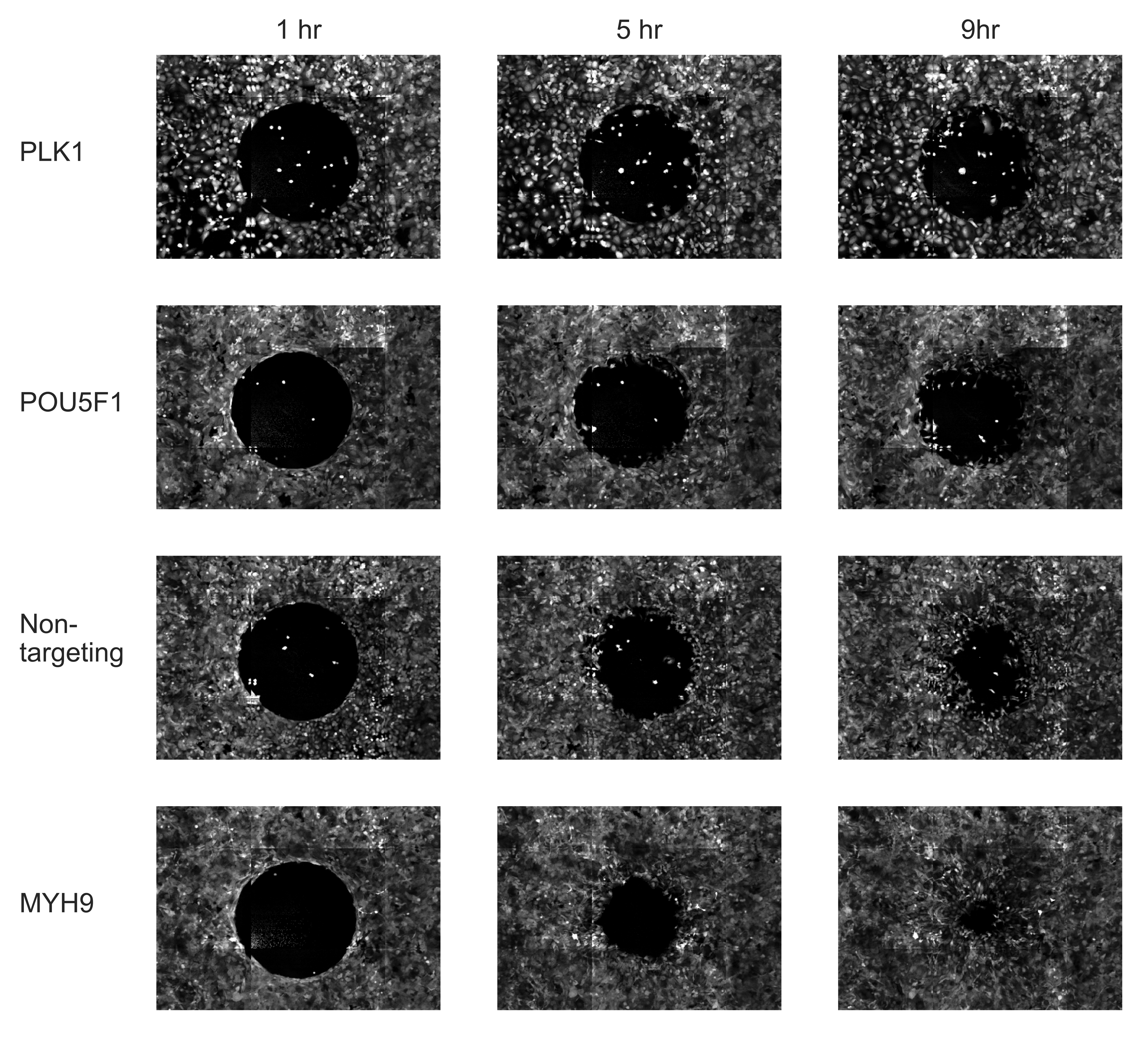

Supplement: S3 Fig — Cells with PLK1 knockdown are not migrating. MYH9 and POU5F1 knockdown cells are migrating faster and slower respectively compared to non-targeting cells. (TIF) [file pcbi.1005900.s003.tif]
